# White Matter Microstructural Damage as an Early Sign of Subjective Cognitive Decline

**DOI:** 10.3389/fnagi.2019.00378

**Published:** 2020-01-28

**Authors:** Caimei Luo, Mengchun Li, Ruomeng Qin, Haifeng Chen, Dan Yang, Lili Huang, Renyuan Liu, Yun Xu, Feng Bai, Hui Zhao

**Affiliations:** ^1^Department of Neurology, Nanjing Drum Tower Hospital, Nanjing University Medical School, Nanjing, China; ^2^Jiangsu Key Laboratory for Molecular Medicine, Nanjing University Medical School, Nanjing, China; ^3^Jiangsu Province Stroke Center for Diagnosis and Therapy, Nanjing, China; ^4^Nanjing Neuropsychiatry Clinic Medical Center, Nanjing, China

**Keywords:** Alzheimer’s disease, subjective cognitive decline, amnestic mild cognitive impairment, diffusion tensor imaging, white matter pathway

## Abstract

**Background and Objective**: Subjective cognitive decline (SCD) is considered a preclinical state of Alzheimer’s disease (AD) and may represent a more advanced preclinical status than amnestic mild cognitive impairment (aMCI). Our aim was to explore changes in the white matter (WM) microstructure and their correlation with cognitive function in these AD-spectrum patients.

**Methods**: Diffusion tensor images from 43 individuals with normal cognition (NC), 38 SCD patients, and 36 aMCI patients were compared using an atlas-based segmentation strategy. The correlation between diffusion parameters and cognitive function was further analyzed.

**Results**: The anatomical pattern of WM impairment was generally similar between SCD and aMCI patients. However, aMCI patients showed significantly lower fractional anisotropy (i.e., corpus callosum forceps major and forceps minor) and increased mean diffusivity [i.e., bilateral anterior thalamic radiation (ATR), left corticospinal tract (CST), forceps minor, left cingulum (cingulate gyrus), left cingulum hippocampus, and left inferior fronto-occipital fasciculus (IFO)] in some tracts than did SCD subjects, indicating a disruption in WM microstructural integrity in the aMCI. Individuals with microstructural disruption in forceps minor, left cingulum (cingulate gyrus), and left cingulum hippocampus tracts performed worse in general cognition and memory function tests, as indicated by line regression analysis.

**Conclusion**: SCD individuals had extensive WM microstructural damage in a pattern similar to that seen in aMCI, although presenting a cognitive performance comparable with that of cognitively healthy individuals. Our results suggest that WM integrity might precede objectively measurable memory decline and may be a potential early biomarker for AD.

## Introduction

Alzheimer’s disease (AD) is a neurodegenerative disorder characterized by progressive cognitive impairment and the loss of daily living abilities, and its pathological processes are thought to occur decades before clinical symptoms were detectable. Amnestic mild cognitive impairment (aMCI), a transitional zone between normal ageing and clinically probable AD, has a dementia conversion rate as high as 10–15% per year (Petersen, 2004). Threatened by the lack of curative treatments for AD and aMCI, advancing the time window for intervention would offer an alternative opportunity to slow down or reverse the deterioration.

Subjective cognitive decline (SCD) refers to the subjective experience of cognitive decline in memory and/or other cognitive functions, without any objective evidence of cognitive impairment (Jessen et al., 2014). Given that elderly people with AD usually experience SCD prior to the onset of dementia, SCD has been regarded as the earliest stage in the continuous progression to AD (Reisberg et al., 2008; Hafkemeijer et al., 2013). Subjective decline in memory vs. in other cognitive domains could result in a higher risk of conversion to subsequent aMCI or AD (Rönnlund et al., 2015; Buckley et al., 2016). A recent review has shown that compared with age-matched controls, individuals with SCD suffered a 4.5-fold higher risk of progressing to subsequent MCI, and the risk of eventual progression to AD increased by 6.5-fold (Lin et al., 2019). Another meta-analysis has suggested that approximately 25% of elderly people with SCD will develop MCI due to AD in the next 4 years (Mitchell et al., 2014). Because, SCD may be the first symptom in the pathological cascade of AD, it is of great value to identify AD-related pathological markers in the SCD population.

Previous studies using cerebrospinal fluid (CSF), structural or functional magnetic resonance imaging (MRI), fluorodeoxyglucose positron emission tomography (FDG-PET), and Pittsburgh compound B-positron emission tomography (PiB-PET) have provided substantial evidence that SCD is linked to underlying AD pathology (Sun et al., 2015; Cheng et al., 2017). For example, decreased amyloid beta protein 42 (Aβ-42) and elevated tau level in CSF were found in patients with SCD (Visser et al., 2009). SCD patients also tend to present AD/aMCI-like gray matter atrophy, decreased metabolism, and increased amyloid load, especially in the hippocampus (Vannini et al., 2017; Zhao et al., 2019).

Although less studied, white matter (WM) degeneration has also been demonstrated as an important feature in the spectrum of AD, as identified by histological examination and neuroimaging studies (Caso et al., 2016). A meta-analysis of diffusion tensor imaging (DTI) research revealed that WM microstructural alterations in aMCI and AD were widespread throughout the brain (Sexton et al., 2011), particularly in direct connections from the ventromedial frontal cortex to the medial temporal lobe (MTL) and from the precuneus to the MTL by the way of retrosplenial cortex (Salat et al., 2010). To a certain extent, these regional tissue changes resembled the anatomic connectivity of MTL structures, which have almost equal effects on memory processes, whereas the executive function alterations are related to the frontal-temporal (especially MTL) WM microstructural damage (Sui and Rajapakse, 2018). Cingulum bundle and fornix are the core WM tracts connecting directly to the MTL. The cingulum bundle is a prominent tract that interconnects the frontal, parietal, and medial temporal sites while also connecting the subcortical nuclei to the cingulate gyrus (Bubb et al., 2018). It is mainly responsible for communications between components of the limbic system, which is vital for normal cognition (Burgel et al., 2006). Impaired integrity of the cingulum microstructure was detected in aMCI and AD, which correlated with memory, attention/executive function, language, and visuo-spatial function impairment (Choo et al., 2010; Wang et al., 2012; Kantarci et al., 2017). Fornix projects from the hippocampus to other brain regions and is also responsible for episodic memory loss in older adults with AD and aMCI (Copenhaver et al., 2006). Actually, the pattern of WM alterations seen in DTI was also anatomically concordant with that of gray matter atrophy, initially starting from the limbic bundles (i.e., MTL) and gradually progressing to the temporal lobe, parietal lobe, and frontal lobe WM tracts (Nowrangi et al., 2013; Konukoglu et al., 2016) as the clinical AD disease progresses. Moreover, this pattern of diffusion abnormalities is associated with the neurofibrillary tangle pathology stage, as well as clinical disease severity (Kantarci et al., 2017). Regarding the association between SCD and preclinical AD dementia, we speculated that WM microstructural changes may occur as early as SCD, yet the characteristics remain to be further investigated.

DTI is an advanced MRI quantitative technique for studying the microstructure of WM by utilizing the diffusion motion characteristics of water molecule interactions in tissues. It can sensitively detect the destruction of the WM microstructure such as demyelination, axonal injury, edema, or necrosis (Shaikh et al., 2018). Fractional anisotropy (FA) and mean diffusivity (MD) are two commonly employed diffusion parameters in the analysis of DTI data. FA reflects the directional variation of water molecule diffusion measured in the different directions (Soares et al., 2013; Shaikh et al., 2018), whereas MD represents the average diffusivity (Soares et al., 2013; Shaikh et al., 2018). The FA decrease and MD increase are parallel to the loss of myelin and axonal membranes that restrict random motion of water molecules along the WM tracts. Together, alterations in FA and MD indicate disruption in WM microstructural integrity. AD subjects had lower FA and higher MD within the temporal, parietal, and prefrontal lobes than had cognitively healthy aging adults in a large body of studies (Sexton et al., 2011; Acosta-Cabronero and Nestor, 2014). Therefore, we expect a similar pattern to be found in aMCI and SCD. The purpose of this study was to explore the differences in WM integrity among cognitively normal elderly, aMCI, and SCD and to analyze the correlation between WM integrity and cognitive function.

## Materials and Methods

### Participants

A total of 117 right-handed Chinese Han subjects (43 normal controls and 38 SCD and 36 aMCI patients) were enrolled in this study at the Department of Neurology of the Nanjing Drum Tower Hospital of Nanjing University Medical School from June 2016 to May 2019. This study was approved by the ethics committees of the Nanjing Drum Tower Hospital of Nanjing University Medical School (clinical trial government identifier: NCT01364246), and written informed consent was obtained from each subject prior to participation. All subjects underwent a comprehensive neuropsychological test and 3.0-T whole brain MRI scanning, a routine blood test, and a general medical examination by an experienced neurologist. The cognitive functions of all the subjects were evaluated by an experienced neuropsychologist using the Chinese version of the Mini-Mental State Examination (MMSE) and the Beijing version of Montreal Cognitive Assessment (MoCA; Lu et al., 2011) as general cognitive function screening; the auditory verbal learning test Huashan version (AVLT) as an episodic memory function assessment (Zhao et al., 2012); and the Clinical Dementia Rating (CDR) scale, activities of daily living (ADL) assessment, Hamilton Depression Rating Scale (HAMD), and Hamilton Anxiety Rating Scale (HAMA). The inclusion criteria for normal control group were as follows: (1) no reported cognition complaints; (2) normal MMSE, MoCA, and AVLT scores adjusted for age and education; and (3) CDR score = 0 and ADL score = 8. Patients with SCD were diagnosed based on the research criteria for pre-MCI SCD (Jessen et al., 2014): (1) self-reported experience of continuous decline in memory (within the last 5 years); (2) MMSE, MoCA, and AVLT test scores all within the normal range after adjusting for age and years of education; and (3) CDR score = 0 and ADL score = 8. The Petersen criteria were applied in the diagnosis of aMCI (Petersen, 2004): (1) subjective memory complaint confirmed by an informant; (2) objective memory impairment detected by the MoCA or AVLT (at least 1.5 standard deviations below normative values for age and/or education); (3) preserved general cognitive function (MMSE ≥ 24); (4) CDR score = 0.5; (5) ADL score = 8; and (6) failure to meet the criteria for dementia according to National Institute on Aging and the Alzheimer’s Association (NIA-AA) criteria. Exclusion criteria for all the subjects were as follows: (1) age less than 50 years old; (2) a history of stroke; (3) central nervous system diseases that could cause cognitive decline, including vascular dementia, Parkinson’s disease, epilepsy, central nervous system infection, subarachnoid hemorrhage, or multiple sclerosis; (4) the presence of WM lesions equal to or higher than Fazekas grade II (Wahlund et al., 2001); (5) severe depression (HAMD ≥ 17), anxiety (HAMA ≥ 21), schizophrenia or other mental illness; (6) severe systemic diseases such as heart failure and kidney dysfunction; (7) history of drugs or alcohol abuse; (8) intolerance of MRI examination or inability to complete neuropsychological testing; and (9) other diseases that may affect cognition.

### Image Acquisition

All MRI scans were acquired using 3.0-T scanner [Achieva 3.0 T TX (eight-channel head coil) or 3.0 T Ingenia (32-channel head coil), Philips, Eindhoven, Netherlands] at Nanjing Drum Tower Hospital. Subjects were placed in the supine position. Sagittal T_1_-weighted MR images were performed by a three-dimensional turbo fast echo acquisition with the following parameters: repetition time (TR) = 9.8 ms, echo time (TE) = 4.6 ms, inversion time (TI) = 900 ms, flip angle = 8°, field of view (FOV) = 256 × 256 mm, matrix = 256 × 256, number of slices = 192, and slice thickness = 1 mm. DTI data were obtained using an echo planar imaging (EPI) sequence with the following parameters: in 32 non-collinear directions diffusion encoding (*b* = 1,000 s/mm^2^ for each direction) and one image with no diffusion weighting (*b* = 0 s/mm^2^), TR = 9,154 ms, TE = 55 ms, flip angle = 90°, matrix size = 112 × 112, FOV = 224 × 224 mm, and slice thickness = 2.5 mm. The total scan lasted 13 min. Additionally, axial T_2_-weighted, diffusion-weighted imaging (DWI) sequence, and fluid-attenuated inversion recovery (FLAIR) sequence were collected to detect acute or subacute infarctions and visible WM damage.

### Diffusion Tensor Imaging Processing

**Figure 1:**
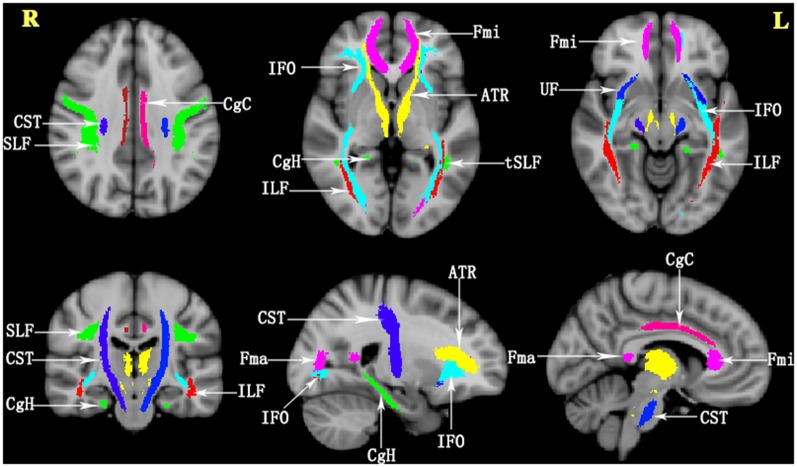
The details of the tracts in the JHU White Matter (WM) Tractography Atlas in the present study. ATR, anterior thalamic radiation; CST, corticospinal tract; CgC, cingulum (cingulate gyrus); CgH, cingulum (hippocampus); Fma, forceps major; Fmi, forceps minor; IFO, inferior fronto-occipital fasciculus; ILF, inferior longitudinal fasciculus; SLF, superior longitudinal fasciculus; UF, uncinate fasciculus; tSLF, superior longitudinal fasciculus-temporal part; L, left; R, right.

The atlas-based segmentation strategy was employed to investigate diffusion abnormalities in this study. The processing of DTI data was carried out with PANDA software, following the default pipeline setting^1^ (Cui et al., 2013). PANDA is a toolbox to perform analyses and calculations of brain diffusion images, which integrates FSL^2^, Diffusion Tool kit^3^, and MRIcron^4^. The major steps include the following: (1) converting original data (DICOM) to the NIFIT format; (2) removing non-brain tissue; (3) correcting eddy current and head motion; (4) adjusting the diffusion gradient direction; and (5) calculating the diffusion tensor metrics, including FA, MD, radial diffusivity, and axial diffusivity. Finally, the FA images in the native space were nonlinearly registered to the FA standard template in the Montreal Neurological Institute (MNI) space using FSL’s FNIRT command. To observe changes in the major WM tracts, all DTI metrics were registered to the JHU WM Tractography Atlas (Hua et al., 2008). Under the present research, only FA and MD diffusion tensor metrics were discussed. The WM fiber pathways of interest were as follows (Figure 1): anterior thalamic radiation (ATR); CST; cingulum (cingulate gyrus; CgC); cingulum (hippocampus; CgH); corpus callosum (forceps major); corpus callosum (forceps minor); inferior fronto-occipital fasciculus (IFO); inferior longitudinal fasciculus (ILF); superior longitudinal fasciculus (SLF); uncinate fasciculus (UF); and superior longitudinal fasciculus-temporal part (tSLF). All of the tracts were evaluated in both hemispheres, except for the corpus callosum (forceps major) and corpus callosum (forceps minor).

### Statistical Analysis

All statistical analyses were performed using the Statistical Package for Social Science (SPSS, v20.0)^5^. The categorization of demographic variables was expressed in terms of frequency and percentage (%), and differences were assessed using the chi-square test. Continuous demographic variables are presented as mean ± standard deviation. One-way analysis of variance (ANOVA) was used to compare the differences in the studied variables (i.e., the results of ADL, HAMA, HAMD, and demographic information) across groups. If the ADL, HAMA, HAMD, and demographic information had significant difference across three groups, *Post hoc* analyses were performed to further explore the details of these group differences using least significant difference (LSD) pairwise comparisons. Furthermore, analysis of covariance (ANCOVA) was used to analyze the results of the cognitive evaluations (MMSE, MoCA, and AVLT) and the diffusion metrics of FA and MD values difference in 20 WM tracts of interest among three groups, with age, gender, and years of education as covariates. Bonferroni correction was conducted to adjust the false-positive rate (*P* < 0.05/20), and significant results further underwent pairwise comparison. To investigate the relationship between the cognitive test scores (MMSE, MoCA, AVLT-immediate recall, AVLT-delayed recall, and AVLT-recognition) and the diffusion parameters of FA and MD, the Pearson correlation analysis was initially performed. The tracts with significance were further included in the stepwise multiple linear regression model, with cognitive scores as dependent variables; FA and MD as independent variables; and age, gender, and years of education as covariates. *P* < 0.05 was considered statistically significant.

## Results

### Demographic and Neuropsychological Characteristics

The demographic information and neuropsychological performance of the three groups are shown in Table 1. There were no significant differences in years of education, gender, vascular risk factors, family history of dementia, and HAMD and HAMA scores among the three groups. However, the NC group was younger overall than the aMCI and SCD groups (it should be noted that the age effect was removed in all subsequent DTI analyses). The comparisons between SCD and NC groups revealed no significant differences in MMSE, MoCA, AVLT-immediate recall, AVLT-delayed recall, or AVLT-recognition scores. General cognitive function (i.e., MMSE and MoCA scores) and memory ability (i.e., AVLT-immediate recall, AVLT-delayed recall, and AVLT-recognition scores) were significantly lower in the aMCI group than in the SCD and NC groups.

**Table 1:** Demographic, clinical, and neuropsychological data.

Variables	NC group (*n* = 43)	SCD group (*n* = 38)	aMCI group (*n* = 36)	*F* or *χ*^2^ value	*P*-value
Gender (F/M)	20/23	21/17	21/15	1.216	0.544
Age (years)	61.91 ± 6.358	66.42 ± 6.685	67.58 ± 6.792	8.342	<0.001^a,b^
Education (years)	12.58 ± 3.417	12.24 ± 3.412	10.86 ± 3.531	2.643	0.076
ADL	8 ± 0	8 ± 0	8 ± 0		
**Vascular risk factors, *n* (%)**					
Hypertension	19 (44.2%)	9 (23.7%)	12 (33.3%)	3.785	0.151
Diabetes mellitus	6 (14.0%)	5 (13.2%)	11 (30.6%)	4.712	0.095
Hyperlipidemia	7 (16.3%)	8 (21.1%)	4 (11.1%)	1.343	0.511
Smoking	4 (9.3%)	2 (13.2%)	2 (5.6%)	1.255	0.534
Drinking	4 (9.3%)	2 (5.3%)	3 (8.3%)	0.494	0.781
Family history of dementia	2 (4.7%)	4 (10.5%)	3 (8.3%)	1.011	0.603
**Mental status**					
HAMD	5.95 ± 4.731	4.66 ± 3.787	5.17 ± 4.45	0.913	0.404
HAMA	8.58 ± 6.318	7.13 ± 6.095	6.08 ± 6.04	1.164	0.198
**General cognition**					
MMSE^#^	28.93 ± 1.183	28.76 ± 1.46	27.47 ± 1.859	4.837	<0.001^a,b^
MoCA^#^	26.7 ± 1.897	26.45 ± 2.076	20.22 ± 2.508	47.641	<0.001^a,b^
**Episodic memory**					
AVLT-IR^#^	17.81 ± 3.756	17.58 ± 4.104	11.94 ± 4.42	10.049	<0.001^a,b^
AVLT-DR^#^	6.09 ± 1.757	5.21 ± 2.183	1.72 ± 1.907	22.531	<0.001^a,b^
AVLT-R^#^	20.98 ± 2.345	20.58 ± 2.815	17.64 ± 3.081	6.985	<0.001^a,b^

*Note. Values are mean ± SD; NC, normal control; SCD, subjective cognitive decline; aMCI, amnestic mild cognitive impairment; AVLT-IR, auditory verbal learning test immediate recall; AVLT-DR, auditory verbal learning test delayed recall; AVLT-R, auditory verbal learning test recognition; ADL, activities of daily living; HAMD, Hamilton Depression Rating Scale; HAMA, Hamilton Anxiety Rating Scale; MMSE, Mini-Mental State Examination; MoCA, Montreal Cognitive Assessment. ^a^*Post hoc* paired comparisons showed significant group differences between NC and aMCI. ^b^*Post hoc* paired comparisons showed significant group differences between SCD and aMCI. ^#^Analysis of covariance (ANCOVA) using age, gender, and education as covariates*.

### Group Comparisons of Atlas-Based Tracts

In general, the diffusion metrics FA and MD in the SCD group were between those of the NC and aMCI groups, as shown in Tables s 2, 3. ANOVA revealed that FA values in the forceps major and forceps minor, as well as MD values in the bilateral ATR, left CST, left CgC, left CgH, forceps minor, and left IFO, were significantly different among the three groups after Bonferroni correction.

**Table 2:** Analysis of covariance of atlas-based tract FA values among the three groups.

Tracts	NC	SCD	aMCI	*F*-value	*P*-value
ATR.L	0.36 ± 0.016	0.343 ± 0.022	0.337 ± 0.031	5.007	0.008**
ATR.R	0.347 ± 0.015	0.327 ± 0.021	0.325 ± 0.028	6.197	0.003**
CTR.L	0.52 ± 0.017	0.508 ± 0.022	0.501 ± 0.025	4.499	0.013*
CTR.R	0.523 ± 0.02	0.512 ± 0.022	0.5 ± 0.027	5.457	0.005**
CgC.L	0.488 ± 0.021	0.47 ± 0.033	0.456 ± 0.035	4.233	0.017*
CgC.R	0.433 ± 0.029	0.414 ± 0.037	0.397 ± 0.04	4.038	0.02*
CgH.L	0.355 ± 0.019	0.341 ± 0.027	0.333 ± 0.029	3.271	0.042*
CgH.R	0.336 ± 0.029	0.325 ± 0.033	0.318 ± 0.033	1.288	0.28
Fma	0.535 ± 0.018	0.512 ± 0.025	0.511 ± 0.03	7.519	<0.001***
Fmi	0.398 ± 0.019	0.377 ± 0.02	0.374 ± 0.025	7.419	<0.001***
IFO.L	0.406 ± 0.017	0.393 ± 0.021	0.387 ± 0.027	3.263	0.042*
IFO.R	0.405 ± 0.018	0.392 ± 0.022	0.388 ± 0.03	2.51	0.086
ILF.L	0.404 ± 0.017	0.391 ± 0.019	0.387 ± 0.023	4.691	0.011*
ILF.R	0.418 ± 0.02	0.409 ± 0.023	0.405 ± 0.023	1.411	0.248
SLF.L	0.36 ± 0.016	0.351 ± 0.017	0.351 ± 0.021	1.538	0.219
SLF.R	0.369 ± 0.018	0.362 ± 0.019	0.359 ± 0.021	0.406	0.667
UF.L	0.367 ± 0.019	0.36 ± 0.027	0.352 ± 0.024	1.208	0.303
UF.R	0.364 ± 0.02	0.358 ± 0.024	0.349 ± 0.028	1.213	0.301
tSLF.L	0.446 ± 0.035	0.447 ± 0.034	0.44 ± 0.039	0.514	0.6
tSLF.R	0.498 ± 0.038	0.494 ± 0.036	0.5 ± 0.042	0.168	0.846

Note. Values are mean ± SD. Analysis of covariance (ANCOVA) using age, gender, and education as covariates. NC, normal control; SCD, subjective cognitive decline; aMCI, amnestic mild cognitive impairment. *P < 0.05; **P < 0.01; ***P < 0.05 after Bonferroni correction (*P* < 0.05/20). ATR, anterior thalamic radiation; CST, corticospinal tract; CgC, cingulum (cingulate gyrus); CgH, cingulum (hippocampus); Fma, forceps major; Fmi, forceps minor; IFO, inferior fronto-occipital fasciculus; ILF, inferior longitudinal fasciculus; SLF, superior longitudinal fasciculus; UF, uncinate fasciculus; tSLF, superior longitudinal fasciculus-temporal part; L, left; R, right; FA, fractional anisotropy.

**Table 3:** Analysis of covariance of atlas-based tract MD values among the three groups.

Tracts	NC	SCD	aMCI	*F*-value	*P*-value
ATR.L	0.836 ± 0.061	0.924 ± 0.127	0.971 ± 0.149	7.658	0.001***
ATR.R	0.843 ± 0.059	0.959 ± 0.132	0.975 ± 0.173	7.899	0.001***
CTR.L	0.774 ± 0.019	0.8 ± 0.033	0.803 ± 0.038	7.103	0.001***
CTR.R	0.779 ± 0.023	0.8 ± 0.033	0.8 ± 0.042	1.738	0.181
CgC.L	0.747 ± 0.024	0.773 ± 0.038	0.781 ± 0.037	6.805	0.002***
CgC.R	0.739 ± 0.024	0.758 ± 0.034	0.761 ± 0.041	1.781	0.173
CgH.L	0.76 ± 0.03	0.793 ± 0.041	0.818 ± 0.072	8.088	0.001***
CgH.R	0.783 ± 0.048	0.821 ± 0.057	0.844 ± 0.135	2.458	0.090
Fma	0.872 ± 0.063	0.925 ± 0.089	0.9 ± 0.099	2.275	0.108
Fmi	0.842 ± 0.035	0.886 ± 0.047	0.909 ± 0.059	16.472	<0.001***
IFO.L	0.779 ± 0.021	0.809 ± 0.04	0.819 ± 0.047	6.589	0.002***
IFO.R	0.788 ± 0.024	0.814 ± 0.043	0.814 ± 0.055	1.728	0.182
ILF.L	0.788 ± 0.022	0.806 ± 0.028	0.814 ± 0.065	1.552	0.216
ILF.R	0.71 ± 0.231	0.726 ± 0.218	0.762 ± 0.141	0.093	0.911
SLF.L	0.721 ± 0.235	0.761 ± 0.229	0.807 ± 0.145	0.217	0.805
SLF.R	0.714 ± 0.232	0.748 ± 0.225	0.788 ± 0.144	0.123	0.884
UF.L	0.737 ± 0.241	0.767 ± 0.232	0.849 ± 0.189	0.817	0.444
UF.R	0.738 ± 0.247	0.771 ± 0.236	0.844 ± 0.201	0.527	0.592
tSLF.L	0.699 ± 0.228	0.708 ± 0.213	0.748 ± 0.133	0.131	0.877
tSLF.R	0.706 ± 0.231	0.713 ± 0.217	0.731 ± 0.135	0.124	0.883

Note. Values are mean ± SD reflecting the MD values × 10^−3^ mm^2^ s^−1^. Analysis of covariance (ANCOVA) using age, gender, and education as covariates. NC, normal control; SCD, subjective cognitive decline; aMCI, amnestic mild cognitive impairment. ***P < 0.05 after Bonferroni correction (*P* < 0.05/20). ATR, anterior thalamic radiation; CST, corticospinal tract; CgC, cingulum (cingulate gyrus); CgH, cingulum (hippocampus); Fma, forceps major; Fmi, forceps minor; IFO, inferior fronto-occipital fasciculus; ILF, inferior longitudinal fasciculus; SLF, superior longitudinal fasciculus; UF, uncinate fasciculus; tSLF, superior longitudinal fasciculus-temporal part; L, left; R, right; MD, mean diffusivity.

**Figure 2:**
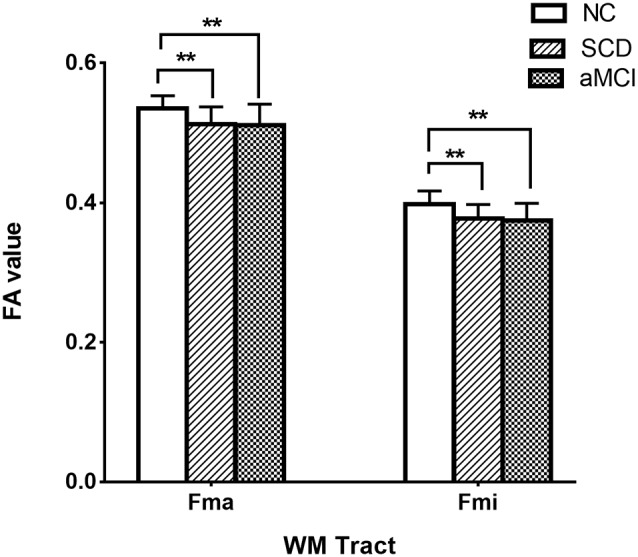
*Post hoc* analysis of FA values for tracts that remained significant after Bonferroni correction in the three groups. NC, normal control; SCD, subjective cognitive decline; aMCI, amnestic mild cognitive impairment; Fma, forceps major; Fmi, forceps minor; WM, white matter; FA, fractional anisotropy; **P < 0.01.

**Figure 3:**
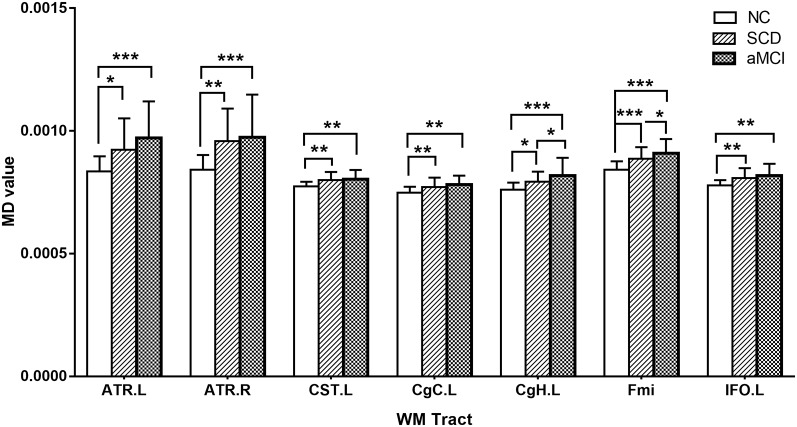
*Post hoc* analysis of MD values for tracts that remained significant after Bonferroni correction in the three groups. NC, normal control; SCD, subjective cognitive decline; aMCI, amnestic mild cognitive impairment; ATR, anterior thalamic radiation; CST, corticospinal tract; CgC, cingulum (cingulate gyrus); CgH, cingulum (hippocampus); Fmi, forceps minor; IFO, inferior fronto-occipital fasciculus; MD, mean diffusivity; L, left; R, right; WM, white matter; *P < 0.05; **P < 0.01; ***P < 0.001.

*Post hoc* comparisons showed that the FA values in the forceps major and forceps minor were significantly decreased in both the SCD group and aMCI group relative to those in the NC group (Figure 2; Table 4), whereas MD values were significantly higher in more extensive regions, including in the bilateral ATR, left CST, left CgC, left CgH, forceps minor, and left IFO (Figure 3; Table 4). However, the SCD group, when compared with the aMCI group, presented significantly lower MD values in the left CgH and forceps minor (Figure 3; Table 4).

**Table 4:** *Post hoc* analysis of the significant tracts surviving Bonferroni correction in the three groups.

White tracts	Group			ANCOVA		*Post hoc*	*P*-value	
	NC	SCD	aMCI	*F*-value	*P*-value	SCD vs. NC	aMCI vs. NC	SCD vs. aMCI
**ATR.L**								
MD	0.836 ± 0.061	0.924 ± 0.127	0.971 ± 0.149	7.658	**0.001**	**0.014**	**<0.001**	NS
**ATR.R**								
MD	0.843 ± 0.059	0.959 ± 0.132	0.975 ± 0.173	7.899	**0.001**	**0.001**	**<0.001**	NS
**CST.L**								
MD	0.774 ± 0.019	0.8 ± 0.033	0.803 ± 0.038	7.103	**0.001**	**0.002**	**0.001**	NS
**CgC.L**								
MD	0.747 ± 0.024	0.773 ± 0.038	0.781 ± 0.037	6.805	**0.002**	**0.008**	**0.001**	NS
**CgH.L**								
MD	0.76 ± 0.03	0.793 ± 0.041	0.818 ± 0.072	8.088	**0.001**	**0.034**	**<0.001**	**0.04**
**Fma**							
FA	0.535 ± 0.018	0.512 ± 0.025	0.511 ± 0.03	7.519	**<0.001**	**0.001**	**0.002**	NS
**Fmi**								
FA	0.398 ± 0.019	0.377 ± 0.02	0.374 ± 0.025	7.419	**<0.001**	**0.001**	**0.001**	NS
MD	0.842 ± 0.035	0.886 ± 0.047	0.909 ± 0.059	16.47	**<0.001**	**<0.001**	**<0.001**	**0.018**
**IFO.L**								
MD	0.779 ± 0.021	0.809 ± 0.04	0.819 ± 0.047	6.589	**0.002**	**0.009**	**0.001**	NS

*Note. Data are represented as mean ± SD; the MD data equal to MD values × 10^−3^ mm^2^ s^−1^. *P*-values in bold indicated meeting statistical significance. NC, normal control; SCD, subjective cognitive decline; aMCI, amnestic mild cognitive impairment; ATR, anterior thalamic radiation; CST, corticospinal tract; CgC, cingulum (cingulate gyrus); CgH, cingulum (hippocampus); Fma, forceps major; Fmi, forceps minor; IFO, inferior fronto-occipital fasciculus; L, left; R, right; FA, fractional anisotropy; MD, mean diffusivity; NS, no significance*.

### Relationship Between Diffusion Metrics and Neuropsychological Scores

The linear regression analysis results between cognitive function scores and FA values in the overall sample are presented in Table 5. In our study, the FA value in the left CgH was independently positively correlated with MMSE (*β* = 0.335, *P* < 0.001) and AVLT-immediate recall (*β* = 0.275, *P* = 0.002) scores; the FA values in the left CgC were independently correlated with performance on MoCA (*β* = 0.295, *P* < 0.001), AVLT-delayed recall (*β* = 0.339, *P* < 0.001), and AVLT-recognition (*β* = 0.296, *P* < 0.001) assessments.

**Table 5:** Multiple linear regression models for different cognitive scores and correlated FA variables.

Dependent variable	Variables included in the model	Unstandardized coefficients *B*	Standardized coefficients *β*	*P*-value	95% confidence interval
MMSE	Constant	19.204		<0.001	14.735–23.672
	CgH.L	26.186	0.335	**<0.001**	13.157–39.214
MoCA	Constant	3.345		0.480	−6.009 to 12.698
	CgC.L	35.868	0.295	**<0.001**	16.231–55.506
	Education	0.335	0.286	0.001	0.146–0.525
AVLT-IR	Constant	−2.519		0.658	−13.764 to 8.726
	CgH.L	53.077	0.275	**0.002**	20.292–85.863
AVLT-DR	Constant	−8.791		0.008	−15.226 to −2.357
	CgC.L	27.843	0.339	**<0.001**	14.167–41.520
AVLT-R	Constant	5.449		0.184	−2.630 to 13.528
	CgC.L	30.015	0.296	**<0.001**	12.843–47.187

*Note. First, all FA values of all atlas-based tracts were correlated with neuropsychological scores, and only variables correlated with neuropsychological score at *P* < 0.05 were used in subsequent stepwise multiple linear regressions controlling for age, gender, and years of education. *P*-values in bold indicated that the corresponding tracts were significantly correlated with cognitive function. AVLT-IR, auditory verbal learning test immediate recall; AVLT-DR, auditory verbal learning test delayed recall; AVLT-R, auditory verbal learning test recognition; MMSE, Mini-Mental State Examination; MoCA, Montreal Cognitive Assessment; CgC, cingulum (cingulate gyrus); CgH, cingulum (hippocampus); L, left; R, right; FA, fractional anisotropy*.

In addition, the results of the regression analysis of the cognitive function scores and the MD values within the entire sample are shown in Table 6. MD values in the left CgH were independently negatively correlated with MMSE (*β* = −1.89, *P* = 0.04) and MoCA (*β* = −0.227, *P* = 0.015) scores. MD values in the left CgC were negatively correlated with MMSE (*β* = −0.193, *P* = 0.037) and AVLT-recognition (*β* = −0.377, *P* < 0.001) scores. MD values in the forceps minor were independently correlated with MoCA (*β* = −0.245, *P* = 0.009), AVLT-immediate recall (*β* = −0.337, *P* < 0.001), and AVLT-delayed recall (*β* = −0.439, *P* < 0.001) scores.

**Table 6:** Multiple linear regression models for different cognitive scores and correlated MD variables.

Dependent variable	Variables included in the model	Unstandardized coefficients *B*	Standardized coefficients *β*	*P*-value	95% confidence interval
MMSE	Constant	40.099		<0.001	32.876–47.323
	CgH.L	−7,055.505	−1.89	**0.040**	−13,771.278 to −339.732
	Education	0.121	0.2	0.02	0.02–0.223
	CgC.L	−10,117.516	−0.193	**0.037**	−19,596.560 to −638.47
MoCA	Constant	47.183		<0.001	36.827–57.540
	CgH.L	−16,324.704	−0.227	**0.015**	−29,484.453 to −3,164.955
	Education	0.381	0.325	<0.001	0.2–0.562
	Fmi	−16,525.910	−0.245	**0.009**	−28,933.617 to −4,118.203
AVLT-IR	Constant	37.190		<0.001	24.546–49.834
	Fmi	−29,064.516	−0.337	**<0.001**	−43,295.34 to −14,833.68
	Education	0.345	0.231	0.006	0.099–0.592
AVLT-DR	Constant	21.965		<0.001	15.543–28.387
	Fmi	−20,011.718	−0.439	**<0.001**	−27,260.017 to −12,763.420
AVLT-R	Constant	44.019		<0.001	33.349–54.69
	CgC.L	−31,774.021	−0.377	**<0.001**	−45,602.9 to −17,945.13753

*Note. First, all MD values of all atlas-based tracts were correlated with neuropsychological scores, and only variables correlated with neuropsychological score at *P* < 0.05 were used in subsequent stepwise multiple linear regressions controlling for age, gender, and years of education. *P*-values in bold indicated that the corresponding tracts were significantly correlated with cognitive function. AVLT-IR, auditory verbal learning test immediate recall; AVLT-DR, auditory verbal learning test delayed recall; AVLT-R, auditory verbal learning test recognition; MMSE, Mini-Mental State Examination; MoCA, Montreal Cognitive Assessment; CgC, cingulum (cingulate gyrus); CgH, cingulum (hippocampus); Fmi, forceps minor; L, left; R, right; MD, mean diffusivity*.

## Discussion

In the present study, changes in the microstructural integrity of WM in AD-spectrum patients were revealed using DTI metrics. The anatomical pattern of WM impairment was similar in both SCD and aMCI patients, and the damage was less severe in SCD patients than in aMCI patients. In addition, significant correlations between diffusion metrics and cognition suggested that those individuals with WM microstructural disruption performed worse in general cognition and memory function tests. These results supported the previous theory that the DTI index can be a sensitive measure for detecting changes of WM microstructural in AD degenerative processes and can offer a new insight into AD pathophysiology (Caso et al., 2016).

Research interest is shifting to increasingly earlier stages, as the origin of AD and keys to treatment probably lie in the prevention of progression to a fully fledged disease (Slot et al., 2018). It should be noted that SCD has been shown to be the first clinical manifestation of AD, and there is increasing interest in the study of individuals meeting SCD criteria to assess the progression of this disease in the subjects who have a higher risk of cognitive decline (Sánchez-Benavides et al., 2018). Therefore, it is essential to evaluate SCD participant characteristics related to cognitive complaints. In the present study, SCD subjects exhibited significantly lower FA and significantly higher MD across extensive regions, as expected from previous AD-spectrum studies (Caso et al., 2016). Specifically, individuals with SCD showed an intermediate level of microstructure changes between those seen in NC and aMCI patients, whereas the damaging patterns of WM fibers were similar to those seen in aMCI. These brain regions showing compromised WM integrity were parallel to the results of a recent SCD study by Ohlhauser et al., who also discovered widespread significant impairment in superior and inferior longitudinal fasciculi, fronto-occipital fasciculi, and corpus callosum (Ohlhauser et al., 2019). Some previous studies have detected impaired WM integrity in the MTL in SCD patients than in normal controls using TBSS analysis, which also provided additional evidence for our research results (Wang et al., 2012; Selnes et al., 2013). Our findings demonstrate that distinctive WM integrity alterations can occur early in SCD, although neuropsychological test results of these patients are comparable to those of healthy controls. Thus, DTI indexes can be a sensitive and reliable imaging technique to detect changes in WM microstructure, even in this very early stage of AD. Moreover, the present approach may contribute to the development of strategies to stratify SCD subjects with different risk levels for AD.

The impaired WM pathways (including the ATR, forceps minor, forceps major, CgC, CgH, and IFO) were the tracts connecting earliest affected gray matter structures in AD (i.e., hippocampus, cingulate gyrus, medial prefrontal cortex, and posterior cingulate cortex). These findings are in line with previous studies reporting decreased FDG metabolism, increased Aβ deposition, and pathological tau accumulation in these regions in SCD, which closely coincided with the imaging features found in early AD patients (Snitz et al., 2015; Buckley et al., 2017; Vannini et al., 2017). In that sense, our findings showing altered WM tracts connecting these main cortical hubs of cognitive brain networks may provide novel insights into the pathological mechanisms of AD from the WM neurodegeneration perspective. Furthermore, most of the mentioned tracts link brain areas belonging to the default mode network, which has consistently been found to be affected across the development of AD (Zhang et al., 2010). Regions in the default mode network, such as the medial prefrontal cortex, are related to working memory, whereas MTL regions are involved in sustaining long-term memory. Many studies have demonstrated that individuals with SCD show abnormal activity in the default mode network, as assessed by functional MRI (Rodda et al., 2009, 2011). Thus, the damaged WM tracts linking the default mode network could be partly responsible for these functional changes.

In addition, the present aMCI group showed significantly higher MD values in the left CgH and forceps minor than in those of SCD patients. These results were in line with the findings by Parra et al., whose participants were young presymptomatic presenilin 1 mutation carriers. These young hereditary AD carriers showed a significant increase in MD values in bilateral CgH and forceps minor (Parra et al., 2015). Jung et al. (2015) also found that in the aMCI group, compared with the SCD group, only the MD value in the left CgH was significantly increased. Therefore, we speculate that the destruction of the WM integrity in the left CgH and forceps minor may be crucial for progressive cognitive impairment. A significant of numerical increases was also observed in MD of the left CgH and forceps minor in SCD and aMCI groups compared with the NC.

The multiple general linear regression model analysis showed that after age, gender, and years of education were adjusted, FA and/or MD values in the left CgC, left CgH, and forceps minor were independently correlated with overall cognitive function and memory function. The lower the FA value and the higher the MD value were, the worse the cognitive performance. The cingulum, as the core structure in the Papez circuit of the cholinergic system, is an important pathway to maintain communication among the limbic system, which plays a role in perception, executive control, episodic memory, and understanding (Bubb et al., 2018). The disruption of the cingulum can lead to the disruption of communication from the hippocampus and cingulate gyrus to the cerebral cortex, subsequently impairing memory and other cognitive domains. The forceps minor connects the bilateral prefrontal cortex, where the encoding and extraction of episodic memory occur (Jeong et al., 2015). Grambaite et al. (2010) noticed in SCD and aMCI subjects who had decreased FA values in the forceps minor were related to verbal memory. Parra’s study found that an increase in the MD values in the forceps minor and left CgH could predict poor short-term memory task performance (Parra et al., 2015), which agreed with our results. Hence, we can infer that WM microstructural impairment may be an important predictor of overall cognitive and memory function and an early marker of the development of AD.

There are still some limitations to this study. First, this study is cross-sectional and is insufficient for predicting the clinical outcomes of SCD and aMCI patients. Second, the present sample of SCD and aMCI participants was recruited based on clinical criteria, which may not have displayed the underlying AD pathology. Future studies should add biomarker information to better characterize the findings. Therefore, these data should be interpreted with caution. Third, WM microstructures may be different owing to the diverse stages of SCD, but currently, there are no clear criteria to classify the severity of SCD. In addition, our study focused only on the changes in the WM microstructure of SCD patients. It is unclear whether these changes are related to gray matter atrophy, functional changes, or CSF pathological indicators. Finally, we only focused on the most commonly used metrics (FA and MD), but other metrics in DTI, such as radial and axial diffusivity, will be investigated subsequently. The longitudinal change pattern of WM microstructure from preclinical to clinical AD needs to be confirmed by large sample longitudinal studies.

In summary, SCD individuals had extensive WM microstructural damage in a pattern similar to that seen in aMCI. DTI-based diffusion parameters could be useful imaging biomarkers for the early diagnosis and stage monitoring of AD. Considering that individuals with SCD are at high risk for aMCI and AD, future studies are needed to characterize individuals with SCD longitudinally to identify individuals who may eventually develop AD, enabling more timely interventions.

## Data Availability Statement

The datasets generated for this study are available on request to the corresponding author.

## Ethics Statement

The studies involving human participants were reviewed and approved by the ethics committees of the Affiliated Drum Tower Hospital of Nanjing University Medical School (clinical trials government identifier: NCT01364246). The patients/participants provided their written informed consent to participate in this study. Written informed consent was obtained from the individual(s) for the publication of any potentially identifiable images or data included in this article.

## Author Contributions

HZ and FB designed the study, collected the data, and edited the manuscript. YX designed the study and edited the manuscript. CL collected the data, wrote and edited the manuscript, and performed the statistics. RQ wrote and edited the manuscript. ML, DY, LH, and RL collected the data. HC validated the statistics.

## Conflict of Interest

The authors declare that the research was conducted in the absence of any commercial or financial relationships that could be construed as a potential conflict of interest.

## Footnotes

^1^http://www.nitrc.org/projects/panda/

^2^https://fsl.fmrib.ox.ac.uk/fsl/fslwiki

^3^http://www.trackvis.org/dtk/

^4^https://www.nitrc.org/projects/mricron

^5^http://www-01.ibm.com/software/analytics/spss
